# A Design and Simulation of the Opportunistic Computation Offloading with Learning-Based Prediction for Unmanned Aerial Vehicle (UAV) Clustering Networks †

**DOI:** 10.3390/s18113751

**Published:** 2018-11-02

**Authors:** Rico Valentino, Woo-Sung Jung, Young-Bae Ko

**Affiliations:** 1 Department of Computer Engineering, Ajou University, Suwon 16499, Korea; ricovalentino94@ajou.ac.kr; 2 Electronics and Telecommunications Research Institute (ETRI), Daejeon 34129, Korea; woosung@etri.re.kr

**Keywords:** drone cluster, computation offloading, neural network, wireless communication

## Abstract

Drones have recently become extremely popular, especially in military and civilian applications. Examples of drone utilization include reconnaissance, surveillance, and packet delivery. As time has passed, drones’ tasks have become larger and more complex. As a result, swarms or clusters of drones are preferred, because they offer more coverage, flexibility, and reliability. However, drone systems have limited computing power and energy resources, which means that sometimes it is difficult for drones to finish their tasks on schedule. A solution to this is required so that drone clusters can complete their work faster. One possible solution is an offloading scheme between drone clusters. In this study, we propose an opportunistic computational offloading system, which allows for a drone cluster with a high intensity task to borrow computing resources opportunistically from other nearby drone clusters. We design an artificial neural network-based response time prediction module for deciding whether it is faster to finish tasks by offloading them to other drone clusters. The offloading scheme is conducted only if the predicted offloading response time is smaller than the local computing time. Through simulation results, we show that our proposed scheme can decrease the response time of drone clusters through an opportunistic offloading process.

## 1. Introduction

Unmanned Aerial Vehicles (UAVs), or drones, have gained much popularity in various applications [1,2,3], including civilian and military applications. Undoubtedly, lower operational cost and higher human safety are the main reasons for this boost in popularity. For example, UAVs are used in several areas, including mail delivery, safety monitoring, smart cities, transport management, and even disaster management [4]. In military application, drones are deployed for surveillance system [5] and enemy engagement [6]. Moreover, the importance of drone deployment has been shown recently from atmospheric applications point of view. UAVs or drones are utilized for monitoring trace tropospheric gases [7]. As global emissions continue to be higher, it is needed a technology that could detect trace gases accurately. Thus, the utilization of UAVs is proposed to gather information about trace tropospheric gases. In [8], small drones are deployed to get enhanced atmospheric physics measurements focusing on atmospheric sampling of thermodynamic parameters. UAV experiment for real-time monitoring of Nanjing’s air pollution is conducted in [9], given that UAV has cheaper deployment cost than manned aircraft. Also, the feasibility of UAVs utilization for measuring turbulence of atmospheric boundary level is proposed [10].

Recently, the deployment of multiple small UAVs as a cluster [11,12] or swarm to execute a variety of tasks has gained more attention as it improves on the effectiveness of the current single UAV system and has a lower cost. Operating a group of small drones rather than a single huge UAV has abundant benefits, which include a guaranteed reliable ad-hoc network, improved operational performance, extended mission coverage [13], and reduced possibility of detection by the enemy in a reconnaissance mission. The tasks of drones are continuously growing larger and more complex despite drones’ limited computing power and energy resources. Thus, it is harder for drones to complete these resource hungry applications in a timely fashion. The problem of insufficient computing resources and energy can be addressed using a computational offloading technique [14,15,16] to a nearby ground control station (GCS), which has unlimited energy and powerful computational power. Alternatively, offloading could be done to neighboring drones that have sufficient resources to process the given task. However, when too many drones offload their workloads to a single GCS, there will be a bottleneck in the GCS, resulting in a longer response time for application execution. Intuitively, the problem can be resolved by providing more powerful GCSs around the drones’ operating site. However, drones services are often required in remote areas, which lack in network infrastructures. In addition, in a disaster area where the communication network has collapsed or in a military operation area, it is very difficult to deploy more powerful GCSs. Therefore, to address the problem from another perspective, assuming that there will be many more deployments of clusters of drones in the near future, we propose an offloading mechanism that is not only done between drones and a GCS, but one that can be done between different clusters of drones. A UAV cluster that requires higher computing power may borrow its neighboring cluster’s idle resources for executing its own task.

In this paper, we consider the feasibility of applying a computation offloading technique to an inter-cluster of drones. More specifically, we propose an opportunistic computation offloading scheme with a learning-based prediction module between drone clusters. First, the head of the cluster will identify the presence of other drone clusters nearby and it will then communicate whether those clusters have idle computing resources. After that, the cluster head will estimate or predict the response time of processing the workloads locally and compare it with that of offloading workloads to the other clusters. The offloading decision will be made by considering some input parameters, which include available computing power, application input size, computing power required to complete the application, and the communication bandwidth of the wireless network. Furthermore, response time estimation is made by adopting a machine learning technique to improve the accuracy of the decision engine. From the estimated result, the cluster head will make the decision of whether to complete the tasks locally or to offload them to the other cluster. If the estimated response time of the offloading scheme is faster than that of the local response time, then offloading mechanism will be triggered; otherwise, the application will be executed locally inside the cluster.

The contributions of this study are threefold:

- We design an opportunistic computation offloading scheme between clusters of drones with the aim of reducing response time. The cluster head will decide whether to use the proposed offloading scheme based on the decision engine prediction module output.
- We design and build a shallow neural network-based response time prediction module to give a better prediction accuracy when compared to the heuristic algorithm.
- We build a NetworkSimulator-3 (NS-3)-based UAV network simulation system for evaluating the drone wireless communication scheme with a mobility scenario.

**Figure 1:**
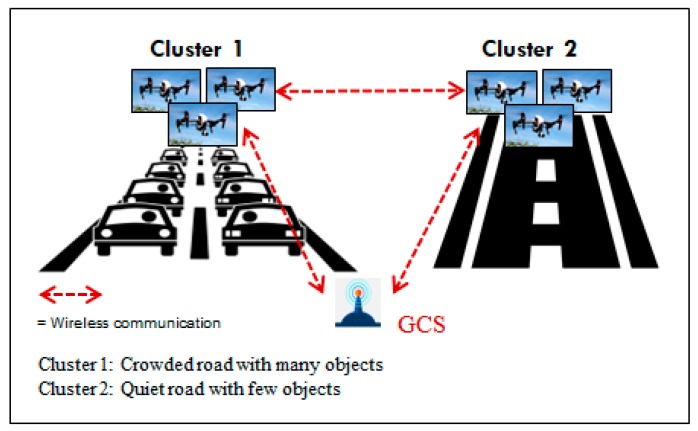
Drone clusters with identical service but different environmental conditions. GCS: Ground Control Station.

Figure 1 shows the scenario of UAV clusters have identical services but different environmental conditions. Say that two different clusters of UAVs make up a surveillance system over two different coverage areas with the similar workload size. One cluster works in a harsher environment (e.g., highly populated road) with many noise objects; the other cluster operates in a convenient environment (e.g., quiet road) nearby. Due to different environmental conditions, the first cluster will have relatively more work and require more time to complete its tasks. The other cluster will finish its task easily without additional burdens. When considering such a situation, the cluster that is deployed in the quiet road will complete its work first. Thus, it will have available computing power and resources that can be borrowed by the other UAV cluster. In other words, the first cluster has a chance to utilize this opportunity to offload some parts of its tasks to the other UAV cluster. Furthermore, since more clusters of drones will be deployed in various application services, the proposed scheme can also be utilized between clusters of drones with different services. For example, this scenario can happen when mail delivery drones fly close enough to a surveillance drone cluster. The surveillance drones may take advantage of this opportunity to get more computing resources to help them finish their work by sending an offloading request. After receiving a reply message, the surveillance drone cluster head may decide whether to conduct the offloading scheme. This scenario may be also adopted by other drone service scenarios.

The remainder of this paper is structured as follows. We summarize the motivation of this work and related work in Section 2. Next, we present our opportunistic computational offloading scheme in Section 3. Then, we show the simulation results in Section 4. Finally, we conclude this work in Section 5.

## 2. Related Works

The concept of computational offloading has been studied for many years. In [17], Loke investigated the possibility of handing off computing tasks to other devices via communication interfaces. In [18], Kovachev et al. proposed an adaptive computation offloading middleware for mobile devices to offload their computation tasks to a cloud server. The results show that the local execution time can be reduced significantly through computation offloading. Deep reinforcement learning-based computation offloading and resource allocations for mobile edge computing (MEC) has recently also been studied in [19]. By adopting a machine learning technique, Li et al. [19] aim to optimize the offloading decision and computational resource allocation for multiple user equipments (UEs) that are trying to access the offloading service of a MEC server. However, when compared to mobile devices, drones have a more dynamic environment, which makes it harder to predict the response time of drones.

Offloading schemes for UAV systems have been developed recently. In [20], Jung et al. designed a computation offloading system in surveillance drone to a GCS. Adaptive Computation Offloading for Drone Surveillance System (ACODS) architecture is proposed in this work. By considering computational cost, I/O cost, and networking cost, the ACODS system compares the delay that is required to complete UAV jobs between being processed in UAV itself or in GCS. Then, the ACODS system will adaptively determine whether to conduct an offloading scheme based on the decision engine result. Ouahouah et al. in [21] proposed a computation offloading scheme among UAVs that conducts internet of things (IoT) tasks. The offloading scheme is done among UAVs. The goals are to enhance the UAVs’ lifetimes and reduce their response times; the solutions are modeled using linear integer programming. Motlagh et al. [22] proposed the offloading of a UAV-based IoT platform’s workload to an MEC node in a crowd surveillance use case. The goal is to reduce the processing time of recognition and detection. A mobility-aware computation offloading decision scheme is proposed in [23]. Based on the mobility information of the moving target object and network conditions, it will offload some computation task that is related to the recognizing and tracking of a moving object to a remote control center. Computation offloading in UAV network was also studied in [24]. Messous et al. [24] addressed the computation offloading decision making problem in order to accomplish intensive computational tasks by adopting a sequential game approach. However, neither of these works address the problem of utilizing an offloading technique between clusters of drones. Moreover, a bottleneck may occur if too many drones offload their task to a single GCS. Therefore, by using our proposed scheme, which offloads the workload between clusters of drones, this bottleneck is resolved.

As for the drone mobility model, Bujari et al. [25] defined some drone scenarios and mobility models. As an overview, the authors categorize a drone’s mobility model as pure randomized, time-dependent, path-planned, group mobility, or topology-control-based. It is important to define the mobility model of drones when simulating drone networks, because we want the drone movements in the simulation to be really similar with the real world drone movements to get reliable results. In our proposed scheme, we utilize path-planned mobility models to fit our simulation scenario.

## 3. Opportunistic Computation Offloading Scheme

**Figure 2:**
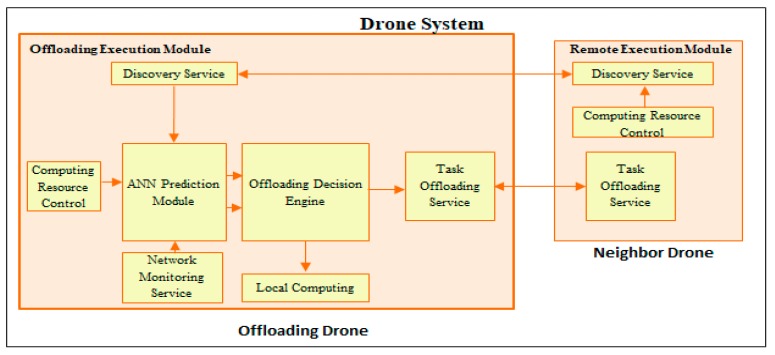
Artificial neural network (ANN)-based opportunistic computation offloading system architecture.

The architecture of the opportunistic computation offloading scheme between clusters of drones is shown on Figure 2. We divide the drone system into offloading drones and neighbor drones. Offloading drones conduct the offloading scheme and neighbor drones are the target for the offloading service execution. In the offloading drone system, there is an offloading execution module, which is the offloading framework to determine the offloading process. Computing resource control is used to monitor the computational resources of the UAV. A network monitoring service is run to obtain the instantaneous data rate of the UAV. A discovery service is used to determine whether the neighbor drone cluster has idle resources. All previous information will be fed into the artificial neural network (ANN)-based prediction module to estimate the response times for executing the application task locally and using the offloading scheme service. The result of the predicted response time will be sent into the offloading decision engine. If the offloading scheme has a shorter estimated response time than the local computing scheme, then the task offloading service will be called to handle the distribution of the workloads to the neighbor drones. Otherwise, the application job will be processed locally within the cluster. A remote execution module is run under the neighbor drone system. It also has a discovery service, which is linked into the computing resource control in order to send information about its available computing power into the offloading request cluster.

### 3.1. Cluster Discovery Scheme

To execute the opportunistic offloading scheme, drone clusters must discover the existence of the other clusters near them first. In the proposed offloading scheme, the fundamental cluster discovery scheme is used. At the beginning phase of the offloading scheme, a cluster with a high intensity task (HIT) that requires the offloading process tells its cluster head to broadcast a discovery message to the nearby environment. The discovery message includes the source cluster ID and a discovery message header.

Drone cluster heads near the HIT drone cluster that received this discovery message will then reply with a message based on their resource utilization situation. If the availability of their computation resources exceeds a certain threshold value, then they will send back a reply message to the cluster head of the offloading requester; otherwise, the message will be ignored. The reply message contains cluster ID and resource utilization information. The HIT drone cluster head will receive the reply message, and then it will save the existence information of nearby drone clusters and their computational resource information to be used further in the next step.

**Figure 3:**
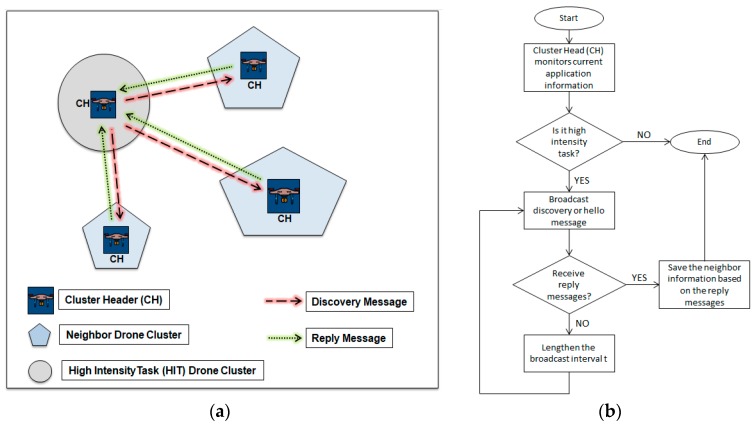
(**a**) Cluster discovery scenario and (**b**) Flowchart of cluster discovery process.

The discovery message itself will be broadcasted periodically by the HIT drone cluster head every *t* seconds. When discovery message has been sent n times with no reply messages received, the broadcast interval *t* will be increased in order to minimize the message overhead. The cluster discovery scenario is shown in Figure 3.

### 3.2. Computation Offloading Decision Module

The cluster head of the local UAV cluster will learn whether there exist other groups of drones after the discovery process is finished. Then, it may be able to proceed to the offloading decision taking stage, which will give a final offloading decision. Final decision will be either to execute the job tasks locally inside the cluster or to do offloading scheme to other clusters. Many kinds of input parameters are considered for taking the offloading decision. The discovery service will provide drone clusters’ presence information and also their computing resources. The streaming image data and other UAV tasks will be obtained from the camera or other sensors. More, the information about vacant computing power on the cluster will be provided by the computing resource control module. Network monitoring service will show the network channel condition between the cluster head and UAV members.

To obtain the final offloading decision, the proposed scheme estimates the elapsed response time (remotely or locally). By comparing whether computing the task locally is faster than offloading to other clusters, the offloading decision engine will come to a final conclusion. In this system, estimation of the response time is done by considering the communication delay (*t_comm_*) and computing delay (*t_comp_*). Let *T* be the estimated time consumed for finishing all tasks.

(1) $$T=t_{comp}+t_{comm}$$

Application tasks are denoted as *A_i_* = (*work_i_*, *Ci*, π*_i_*), where *work_i_* is the input parameter size of application *i*, *C_i_* is the computation cycle required to complete the task, and π*_i_* is the maximum tolerable delay to finish the application task. Computation delay could be calculated by accumulating all the computation power of each UAV cluster member. Computation power is obtained by multiplying the fully utilized central processing unit (CPU) computing delay of one cycle of the task (*t_optimized_*) with the available CPU resources (*r_avail_*) in percentage. CPU resources are usually partially used for stabilizing the drone position in the air and also for processing other software tasks; hence, (*r_avail_*) represents the idle computing resources of the CPU. Given that *N* is the total number of drones in a cluster, the computation delay (*t_comp_*) of each cluster can be written as (2).

(2) $$t_{comp}=\sum_{i=1}^{N}\left(C_{i}\;x\;t_{optimized_{i}}\;x\;\frac{1}{r_{avail_{i}}}\right)$$

Communication delay can be summarized into two types: cluster member to the cluster head (*t_intra_*) within the same cluster and local cluster head to the remote cluster head (*t_inter_*). In predicting the communication time cost, we consider the data rate of the communication network (*BW*). For the *t_intra_* case, the maximum *t_intra_*_(*i*)_ value will be used as the local communication cost, when considering the fact that each member could have a different *t_intra_* value. For *t_inter_*, the total input parameter size of the application task to be offloaded (*work_CH_*) will be divided by the network data rate at that time.

(3) $$t_{intra}=\underset{1\leq i\leq N}{\max}\frac{work_{i}}{BW_{i}}.$$

(4) $$t_{inter}=\frac{work_{CH}}{BW_{CH}}.$$

Therefore, the estimated elapsed time for completing tasks locally (*T_local_*) and doing offloading (*T_remote_*) can be written as (5) and (6), respectively (given that the remote cluster has *j* members).

(5) $$T_{local}=\left[\sum_{i=1}^{N}\frac{C_{i}\;x\;t_{optimized_{i\;}}}{r_{avail_{i}}}\right]+\underset{1\leq i\leq N}{\max}\frac{work_{i}}{BW_{i}}$$

(6) $$T_{remote}=\left[\sum_{j=1}^{N}\frac{C_{j}\;x\;t_{optimized_{j\;}}}{r_{avail_{j}}}\right]+\underset{1\leq j\leq N}{\max}\frac{work_{j}}{BW_{j}}+\frac{work_{CH}}{BW_{CH}}$$

From the estimated response time on (5) and (6), the decision engine is able to decide whether to compute the tasks locally or to offload the job application to the remote UAV cluster. By sharing the task opportunistically, the cluster’s task can be distributed more evenly; therefore, it would likely to increase operation time of each drone because the scheme will prevent usage exploitation of one single cluster only. Borrowing the available computing resources from other clusters would likely decrease the response time of the drones. However, computing the estimated response time heuristically could result in some error on the prediction module engine, which eventually will decrease the offloading performance due to suboptimal decisions. Also, computing the response time estimation every time that the UAV clusters want to finish its task will cause too much delay overhead. Therefore, to enhance the current prediction module performance, we adopt a machine learning technique to learn the optimal offloading decision given various combinations of the task size, network condition, and computation resources of each UAV.

### 3.3. Artificial Neural Network-based Response Time Prediction Module

To achieve a more accurate response time prediction module, we propose an artificial neural network-based prediction module as a part of the decision engine on our system. Unlike the work in [26], which uses a linear regression technique and heuristic algorithm to predict the estimated response time, in this work, the proposed scheme utilizes the machine learning technique, specifically an ANN. The main reason is that the response time value is actually non-linear data, so a higher prediction error would likely to occur if linear regression was used. Instead, by adopting the ANN, we could train and test the network to have a non-linear regression model to better estimate a response time.

**Figure 4:**
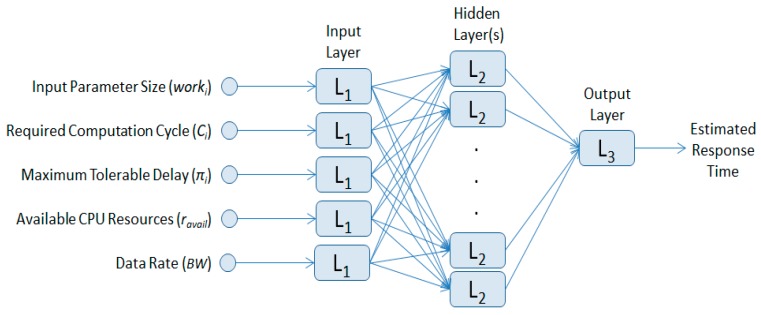
ANN-based response time prediction module.

Typical feed-forward neural networks are composed of an input layer, one or more hidden layers, and a single output layer (see Figure 4). The input layer will include *work_i_*, *Ci*, π*_i_*, *BW*, and *r_avail_* as input features. We use 10 hidden layers to achieve optimal performance for our computation offloading scheme. The optimal number of the hidden layers can be obtained by brute force in the network training session. The number of the hidden layers that will give the best performance will be different as the data input changes; therefore, a validation technique is used to ensure the generality in the trained prediction module. The output layer will give the estimated response time of our offloading scheme. Our proposed scheme adopts an offline learning approach, so we trained our neural network before deploying it to the drone system. By doing so, we only have one time delay cost for training the network beforehand. After being trained, we deploy the ANN-based response time prediction module to each drone cluster.

### 3.4. Task Offloading Service

This service module is responsible for distributing the workload when the cluster head wants to do the offloading process. The task offloading service will be run after the offloading decision is made. After obtaining the information about the destination cluster and its resource information from the offloading request reply message, this module will divide and distribute the workload between the local cluster and the destination remote cluster. All of the workload will be sent through the local cluster head to the destination cluster head. Each cluster head finally takes care of distributing the smaller workload again to each cluster member. After processing the task, the task result is integrated again in the cluster head in a similar way. Since our focus in this study is not on the load distribution algorithm itself, we adopt an existing algorithm in this work [27,28,29].

## 4. Performance Evaluation

### 4.1. Simulation Environment

In this section, we describe the performance evaluation of our proposed scheme while using the NetworkSimulator-3 (NS-3) simulator. Since test-bed implementation is costly and the risk of using drone clusters is very high, performance evaluation using a simulation tool is the best option. The NS-3 simulator was chosen as the main tool for simulating our proposed scheme because its implementation stack is very similar to the real-world network stack. In other words, by implementing and simulating the scheme in NS-3, it is most likely that the implemented scenario will also work well in a real-world environment. Moreover, NS-3 supports mobility models for node mobility, so the UAV mobility model can be implemented more easily.

Main differences between normal ad-hoc nodes and drone nodes can be seen clearly from the computing power model and mobility model. In this study, the computing power model value from each drone is obtained using a reference computing power value as in [20]. Moreover, we utilized path-planned mobility model for UAV clusters to match our simulation scenario by modifying the waypoint mobility model on NS-3.

To the best of our knowledge, utilizing ANN for computational offloading between clusters of UAVs has not been studied in the literature. Thus, our main focus in this performance evaluation is to investigate the response time reduction by comparing our proposed offloading scheme’s response time to the response time for locally completing jobs in the cluster. Also, we focus on error reduction by using an ANN-based response time prediction module to obtain a better result in the final decision engine. In this simulation study, drone clusters perform the proposed scheme in at 40 GHz and 80 GHz of bandwidth. More bandwidth means that the drone cluster networks have a higher chance of achieving a faster data rate to send the workloads. The total application size is also varied from 1–75 MB to demonstrate the performance of the proposed offloading scheme further. Table 1 summarizes the simulation parameters that are used in this study.

### 4.2. Simulation Results

In this section, we discuss the simulation result and analysis. We focus more on the response time parameter for this simulation result as our goal is to reduce the application response time by providing an opportunistic offloading scheme. Recall that response time is a very important factor for UAVs or drones, since they have a relatively short lifetime. Application work size is chosen as the other parameter to show how it affects the proposed offloading scheme’s performance.

**Figure 5:**
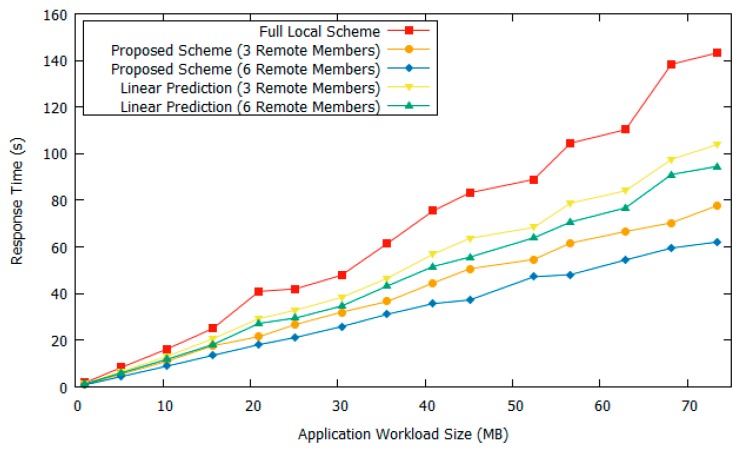
Response time comparison with 80 GHz bandwidth.

Figure 5 shows the response time comparison between the proposed scheme and full local computing when using the 80 GHz network bandwidth. The full local computing scheme executes the application fully within the cluster without considering the offloading scheme. Meanwhile, the proposed scheme’s response time is obtained by performing the offloading scheme according to the offloading decision engine. The learning-based prediction module will predict the estimated response time of the offloading scheme and forward the result to the decision engine; thus, if the estimated offloading response time is faster than with no offloading scheme, the drone will execute the offloading service. In this scenario, the drone cluster that wants to use the offloading scheme selects a nearby targeted cluster. We vary the remote targeted cluster members as three and six drones. A larger cluster means there is a higher chance of more computing resources being available.

The application workload size is varied from 1–75 MB of streaming image data. From Figure 5, it is shown that, by applying our proposed offloading scheme, the drone system can reduce the response time when compared to performing the application task locally. When the chosen remote cluster target had three drone members, our ANN-based offloading scheme reduced the response time by an average of 39% and 20.5% as compared to the fully local computing scheme and linear prediction scheme, respectively. In the linear prediction scheme case, it had higher prediction error probability compared to the ANN-based prediction scheme. This is because when the offloading tasks were supposed to be offloaded to obtain a faster response time, the offloading decision engine decided to do local computing instead because the prediction module result was incorrect. Thus, this scenario degrades the offloading scheme performance. With a better response time prediction module, the ANN-based offloading scheme can obtain better performance.

When the targeted offloading drone cluster had six members, it can be seen that our proposed scheme had a faster response time by 50.7% and 29.3% as compared to the fully local and linear prediction-based scheme, respectively. When some drone clusters wanted to use the offloading scheme, they selected a targeted cluster to offload their tasks. The targeted drone cluster could have various numbers of cluster members. We show that when the targeted cluster has more members, performance is increased. This could happen because having a larger number of cluster members increases the chance of having more computing resources. According to our prediction model, the performance will be increased when the remote drone cluster has more power resources and better network conditions.

**Figure 6:**
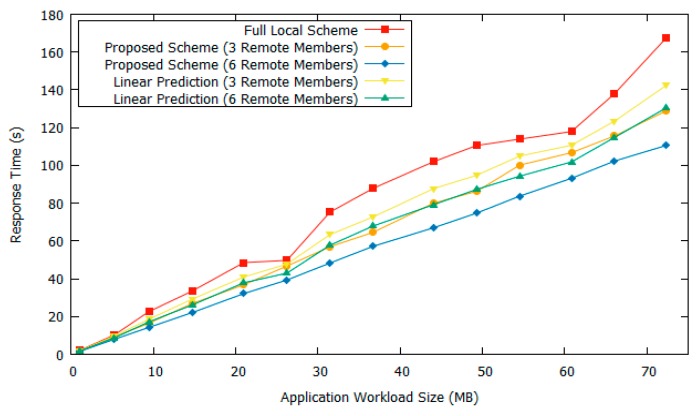
Response time comparison with 40 GHz bandwidth.

In Figure 6, the network bandwidth setting is changed into 40 GHz bandwidth. The instantaneous data rate was smaller as the bandwidth decreased. It can be seen in Figure 6 that the overall performance of our proposed scheme is lower compared to at 80 GHz bandwidth. This is because one of the main factors determining the offloading response time is communication cost. If the application workload size is not too large and the network data rate is small, it will be better to use local computing because of the communication cost of sending the data with the offloading scheme. Our proposed scheme has 18.3% and 30% better performance compared to the fully local scheme when the number of remote members is three and six, respectively. As the network bandwidth decreased, more application tasks were executed locally; therefore, the response time was closer to that of the fully local scheme. Moreover, our proposed scheme had a 7.4% and 13% gain as compared to the linear prediction-based scheme when the targeted cluster had three and six remote members, respectively.

Because the response time data is not linear, using linear prediction for this kind of environment produce relatively more errors. Therefore, in this work, we proposed an ANN-based response time prediction module to predict the non-linear data of response time. In this simulation, we used 10 hidden layers in the ANN network. The number 10 was obtained through a brute force mechanism to train our network to have the best performance results. We adopted the Levenberg-Marquardt [30] optimization algorithm to train our network.

**Figure 7:**
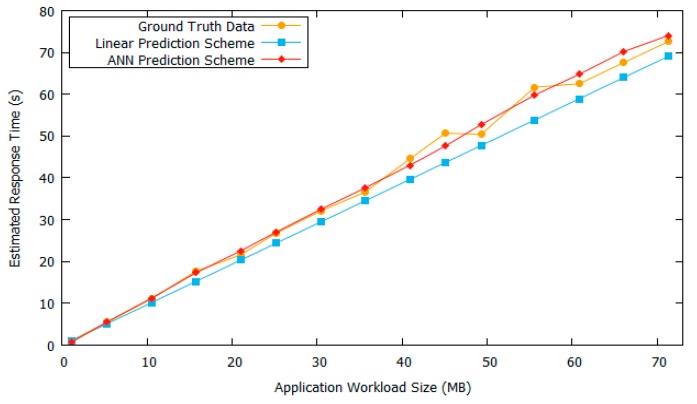
Response time estimation result comparison.

Figure 7 shows the response time estimation result when using both linear prediction and ANN prediction. It can be seen that the ANN prediction provides a closer estimation to the ground truth response time data as compared to linear prediction. Because the ANN-based prediction module uses a non-linear training method, so it follows the ground truth data better.

**Figure 8:**
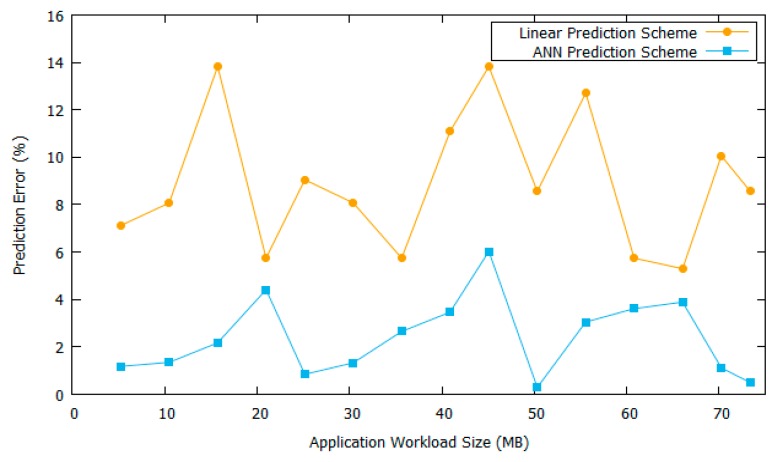
Prediction error graph.

**Figure 9:**
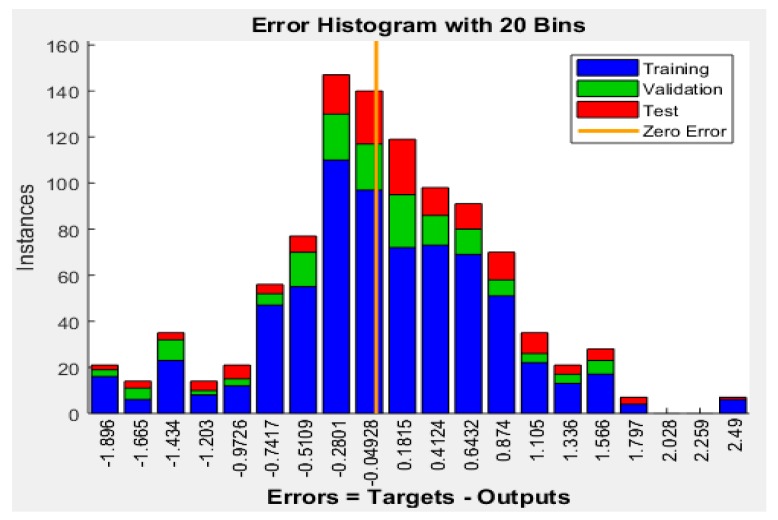
ANN training error histogram.

Figure 8 shows the error graph between ANN prediction and linear prediction. As can be seen in this graph, ANN prediction has a smaller error percentage as compared to the linear prediction. By having a better response time prediction module, the drone clusters system will have a better performance in deciding whether to do the offloading scheme or local computing. Figure 9 shows the ANN training error histogram. We used 70% of data for training, 15% for testing, and 15% for validation. The validation phase was necessary to provide our network with more generality for facing a variety of input data. Moreover, we varied the number of training data from 150 to 1000 data. We found that the average error predictions for 150 and 1000 data are 3.1% and 2.4%, respectively. It shows that our neural network design is able to overcome the generality issue of the input data. It means that even though the total of the training data is small, the performance is not greatly reduced. It is because we use the validation phase on our training effort to provide generality to our network.

**Figure 10:**
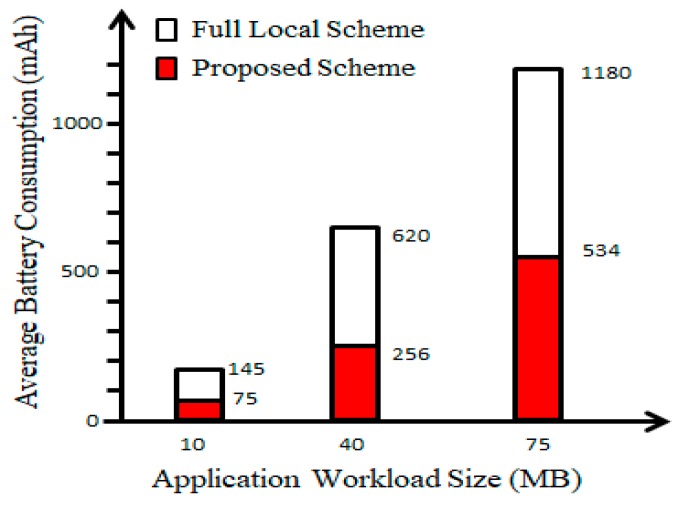
Average battery consumption of full local scheme and proposed scheme.

Because drones have limited power resources, energy consumption becomes a significant issue in terms of drones’ flying time. Energy consumption of drones can be expressed as $E_{i}=z_{i}C_{i}$, where *C_i_* is the computation cycle required to complete the task and *z_i_* represents energy consumption per CPU cycle to complete application task *A_i_*. According to the practical measurement in [31], we set $z_{i}=\;10^{-27}{\left(F\right)}^{2}$ where *F* is the drone computation capacity (i.e., CPU cycles per second). In this simulation experiment, *F* is set to 1 GHz/s. In Figure 10, average battery consumption of full local scheme and the proposed scheme are shown. Our proposed scheme could reduce the average battery consumption on various application workload sizes by around ~50% as compared to the full local scheme. This happens because parts of the application tasks are offloaded to the other drone cluster; therefore, the energy consumption is mostly spent on operating motors in the air only.

## 5. Conclusions and Future Work

Drones are gaining in popularity, and this is proven by the many services that are now provided by drones. Due to the need of more complex workloads as drone technology grows, the utilization of multiple UAVs or drone clusters is preferred because clusters of drones are capable of increasing the coverage area, reducing response time, and offering more reliability than a single UAV. However, drones still have limited battery and computing power; therefore, in order to increase their operating time and reduce their response time, in this study, we proposed a scheme to conduct computational offloading opportunistically between drone clusters. By estimating the elapsed response time of processing tasks locally or remotely, the proposed method can adaptively offload some work to a remote drone cluster. By using the proposed scheme, we can increase drone operation time by distributing the workload more evenly and reduce the response time by borrowing available computing resources from other drone clusters. Moreover, the ANN-based response time prediction was utilized to give the offloading system better performance. In future work, we would like to implement our opportunistic offloading scheme between drone clusters on various real test bed environments and enhance the current offloading algorithm.

## Figures and Tables

**Table 1:** Parameters and values setup for simulation analysis.

Simulation Parameters	Values
Application input size	1–75 MB
MAC & PHY	IEEE802.11ac
Network bandwidth	40 GHz and 80 GHz
Propagation loss model	Three log distance Nakagami fading
Simulation environment	1 km × 1 km
UAV computing power	217.6 ns/Byte
Discovery message interval	5 s

UAV: Unmanned Aerial Vehicles.
